# The Impact of Adverse Childhood Experiences on Asthma Severity in US Adults

**DOI:** 10.3390/medsci12040063

**Published:** 2024-11-11

**Authors:** Chukwuemeka E. Ogbu, Ioannis Stouras, Chisa O. Oparanma, Stella C. Ogbu, Chinazor Umerah

**Affiliations:** 1 Department of Internal Medicine, Cape Fear Valley Health, Fayetteville, NC 28304, USA; 2 Department of Medicine, National and Kapodistrian University of Athens, 11528 Athens, Greece; 3 Department of Medicine, Kharkiv National Medical University, 61022 Kharkiv, Ukraine

**Keywords:** adverse childhood experiences, asthma severity, asthma

## Abstract

**Background/objectives**: The association between adverse childhood experiences (ACEs) and asthma severity among United States (US) adults with asthma has not been well documented. In addition, whether gender differences exist in this association has been underexplored. We aimed to examine the prevalence of asthma severity in the US adult population with asthma and investigate the association between ACEs and asthma severity by using data from non-institutionalized US adults with asthma. **Methods**: This cross-sectional study used data from the Adult 2019 and 2020 Behavioral Risk Factor Surveillance System (BRFSS) Asthma Call-Back Survey (ACBS), a survey of US adults aged 18 years or older in 31 US states and Puerto Rico. A total of 22934 adults with asthma participated in 2019 and 2020 ACBS. The 11 BRFSS ACE variables encompassing abuse and household dysfunction were used as ACE measures. ACE measures were summed up as cumulative ACE scores (continuous) and categorized (zero, one ACE, two ACEs, ≥ three ACEs). Asthma severity was categorized as intermittent or persistent. Weighted logistic regression models were used to assess associations of the cumulative ACE score, categorical ACE measures, and the 11 individual ACE responses with asthma severity controlling confounders. Gender differences were explored by stratifying by gender. **Results:** The prevalence of persistent asthma among US adults with asthma was 45.3%. The mean cumulative ACE score in adults with intermittent vs. persistent asthma was (2.43 vs. 2.70, *p*-value < 0.05). About 22% of adults with asthma had no ACEs, 19% had one ACE, 14% had two ACEs, and 45% had three or more ACEs. A one-unit increase in ACEs score was associated with a 5.4% increase in the odds of persistent asthma (adjusted odds ratio, aOR = 1.054 (95% confidence interval, CI = 1.01–1.10). Experiencing ≥ three ACEs compared to no ACEs was associated with 31% increased odds of persistent asthma (aOR = 1.31, 95% CI = 1.01–1.70). Individual ACE items significantly associated with persistent asthma include parent/adult ever touched you sexually (aOR = 1.33, 95% CI = 1.03–1.74), adult tried to make you touch them (aOR = 1.34, 95% CI = 1.01–1.79), any adult forced you to have sex (aOR = 1.44, 95% CI = 1.04–1.20), parental separation/divorce (aOR = 1.31, 95% CI = 1.05–1.63), and household alcohol abuse (aOR = 1.24, 95% CI = 1.01–1.53). In women, experiencing one ACE and ≥ three ACEs (compared to no ACEs) was associated with 51% and 60% increased odds of persistent asthma, respectively (aOR = 1.51, 95% CI = 1.02–2.23; aOR = 1.60, 95% CI = 1.12–2.27). No significant association was observed between ACEs and asthma severity in men; however, experiencing household physical violence (compared to no household physical violence) was associated with persistent asthma in men (aOR = 1.69, 95% CI = 1.18–2.42). **Conclusions**: In this cross-sectional study of US adults with asthma, exposure to ACEs was associated with higher odds of asthma overall and in women. These findings highlight the importance of preventive strategies and early interventions to reduce ACEs, potentially mitigating asthma’s severity in adulthood.

## 1. Introduction

Asthma is one of the most common non-communicable chronic diseases, affecting over 300 million people globally, among which 25 million reside in the United States [1]. The numbers are expected to increase even more by 2025, according to the World Health Organization (WHO) [2]. The majority of people affected present non-specific symptoms of dyspnea, bursting episodes of wheezing cough, and chest tightness [3]. Although recent studies have shown that asthma is characterized by a significant phenotypic heterogeneity [4], most of the aforementioned symptoms render asthma the disease with the highest rates of school absenteeism [5] and missed workdays [6].

A plethora of risk factors has been shown to confer an association with the onset and/or the severity of asthma (e.g., exposure to tobacco [7], pollutants [8], genetics [9], microbiome [10], etc.) [11]. Over the past three decades, the effort to reveal more risk factors carrying a high risk of asthma onset has shed light on adverse childhood experiences (ACEs). ACEs refer to traumatic events one experiences as a child, including abuse (somatic, verbal, sexual, and emotional). In addition to abuse, the term also includes the parental incapability of supporting a child’s needs, poor socioeconomic status, as well as stressful domestic situations, such as separation, loss of a parent, or illicit substance use [12]. Despite affecting almost half of the United States adult population before the age of 18 years [12], ACEs are still often overlooked and underrecognized in clinical settings.

Evidence tracking back to 1998 supports that ACEs can have a detrimental effect on both mental [13,14] and physical well-being [14] and have been linked to the development/onset of a variety of chronic diseases in adulthood [15], including asthma [16,17,18]. However, data regarding the role ACEs play as a factor in adult asthma severity is limited. Ross et al. [19] have demonstrated that ACEs were significantly associated with a higher degree of parental-reported childhood asthma severity using the National Survey of Children’s Health [19]. However, this study—as the authors have pointed out—is subject to several limitations, including high rates of recall bias, since the parents might not accurately evaluate and report asthma severity. The study occurred in children and is therefore not generalizable to the adult asthma population. In addition to this, asthma severity was evaluated based on symptom evaluation by parents and not according to the necessity of long-term control therapy, which is generally considered a more objective index. Furthermore, there are no studies to date that have evaluated the gender differences in the association of ACEs and asthma severity in the US adult population using population-level data.

This study attempts to address a significant gap in our understanding of factors affecting asthma severity in adults by examining the role of ACEs. Even though the relationship between childhood adversity and asthma onset has been investigated in children, their impact on adult asthma severity, especially considering potential sex differences, remains unknown. Herein, we determined the prevalence of asthma severity in the US adult population with asthma as well as investigated the association between ACEs and adult asthma severity by using data from non-institutionalized US adults with asthma, aiming to provide insights that could influence future therapeutic strategies and public health interventions.

## 2. Materials and Methods

### 2.1. Study Population

The 2019 and 2020 Behavioral Risk Factor Surveillance System (BRFSS) Asthma Call-Back Survey (ACBS) was used for this analysis [20]. The BRFSS, supported by the CDC, is a state-level survey that collects data on chronic diseases, injuries, health behaviors, preventive practices, and healthcare access, including preventable infectious diseases. The ACBS is conducted approximately two weeks after the BRFSS and gathers annual data on asthma, capturing demographic and medical information at both state and local levels. Eligibility for ACBS participation is limited to respondents who report a physician diagnosis of asthma in the BRFSS. In 2019 and 2020, the ACBS response rates as calculated by the Council of American Survey and Research Organization (CASRO) guidelines for participating states, territories, and Washington, D.C., were 49.4% and 49.7%, respectively [21,22].

In 2019 and 2020, 22,934 adults with asthma from 31 states and Puerto Rico participated in the ACBS (11,247 in 2019 and 11,687 in 2020). After excluding individuals with a history of COPD, 19,033 adults with asthma remained in the final sample. Sample weights were adjusted per ACBS guidelines. Relevant state IRBs and the Ethics Review Board of the Asthma and Community Health Branch at the CDC approved the survey. Informed consent was obtained from all participants interviewed in the ACBS. Further details on data, sampling, and analysis methods are available elsewhere [20].

### 2.2. Response Variable: Asthma Severity

The determination of asthma severity adhered to methodologies that align with previous CDC publications that have used the ACBS data to derive population asthma severity estimates [23,24,25].

First, the respondents were classified as experiencing well-controlled, not well-controlled, and very poorly controlled asthma using three measures of impairment, namely: daytime symptoms during the past 30 days, nighttime symptoms during the past 30 days, and use of short-acting beta-2 agonists (SABA) during the past 3 months for symptom control (not for the prevention of exercise-induced bronchospasm) (Supplementary Table S1) [23]. This study used a modified version of the 2007 National Asthma Education and Prevention Program (NAEPP) guidelines, as the ACBS lacked certain measures for current impairment (e.g., pulmonary function) and future risk (e.g., asthma exacerbations or lung function decline) [23,24,25,26].

Secondly, the use of long-term asthma control medications (e.g., inhaled and systemic corticosteroids, long-acting beta-2 agonists, leukotriene receptor antagonists, methylxanthines, and immunomodulators) was ascertained from the reported medication history and categorized as a binary variable (yes or no) [23,24].

Based on the assessment of asthma control status and long-term control medication usage, asthma severity was classified into two categories, namely “intermittent asthma” and “persistent asthma” like in prior studies [23]. Intermittent asthma represents participants with current asthma who are well-controlled without being on long-term control medications. Persistent asthma includes those on control medications, regardless of asthma control status, and those not on long-term control medications whose asthma is not well controlled or is very poorly controlled [23] (Supplementary Table S2).

### 2.3. Explanatory Variable: Adverse Childhood Experiences (ACEs)

The BRFSS ACEs module utilizes an 11-item ACEs scale for public health surveillance [27] (Supplementary Table S3). This measurement encompasses various measures such as physical abuse (parent/adult in your home ever hit, beat, kicked, or physically hurt you in any way), sexual abuse (parent or an adult ever touched you sexually, an adult tried to make you touch them, any adult force you to have sex), emotional abuse (parent/adult in your home ever swear/insult or put you down), witnessing parents’ or caregivers’ intimate partner violence, residing in a household with individuals affected by mental illness, lived with someone with substance abuse issues, had an incarcerated household member, and experienced parental separation or divorce [27]. ACE measures were summed up as cumulative ACE scores (continuous) and categorized (zero, one ACE, two ACEs, ≥three ACEs) [28]. Two composite variables for abuse (physical, emotional, sexual abuse) and household dysfunction (incarcerated, parental separation, household physical violence, substance abuse, mental health, and household alcohol abuse) were also created from the corresponding ACEs items representing them.

### 2.4. Confounders

Confounders included in the analysis were age (18 to 34 years, 35 to 44 years, 45 to 54 years, 55 to 64 years, or ≥65 years), gender (female or male), race (Hispanic, multiracial/other non-Hispanic, white non-Hispanic, Black non-Hispanic), smoking history (current smokers, former smokers, never smokers), household smoke exposure (yes or no), presence of mold in the home (yes or no), household income (≥USD 25,000 or <USD 25,000). Comorbidity status was treated as a continuous variable and was ascertained by summing self-reported health conditions from the BRFSS (diabetes, overweight or obesity, heart attack, angina/coronary artery disease, stroke, skin cancer, depressive disorder, any other cancer diagnosis, arthritis, kidney disease). A systematic review has shown that the total number of comorbidities predicts various health outcomes effectively and performs comparably to more complex measures of disease morbidity [29].

### 2.5. Statistical Analysis

The ACBS used a stratified, multistage survey design to ensure it reflects the U.S. adult population with asthma. Our analysis followed the recommended guidelines. We used PROC SURVEYFREQ and PROC SURVEYLOGISTIC to handle the complex survey data. Sample weights were adjusted for strata, clusters, and primary sampling units to enhance accuracy and reliability. Weighted logistic regression was conducted to examine associations between continuous and categorical ACE variables and asthma severity, adjusting for confounders. Separate models were used to evaluate composite abuse and household dysfunction variables as well as individual ACE items. Gender differences were explored through gender-stratified models, with adjustments made for predictors and confounders, except gender itself. Results were reported as odds ratios (ORs) with 95% confidence intervals (CIs), with proportions as weighted percentages. No multicollinearity was detected, confirmed by tolerance values above 0.1 and VIFs below 10 [30]. Statistical significance was determined by *p* < 0.05 or non-overlapping 95% CIs. Data analysis was conducted using SAS 9.4 (SAS Institute, Cary, NC, USA)

## 3. Results

### 3.1. The Prevalence of Asthma Severity and ACEs

The weighted prevalence of persistent asthma among US adults with asthma was 45.3% (Table 1).

The mean cumulative ACE score in adults with intermittent vs. persistent asthma was (2.43 vs. 2.70, *p*-value < 0.05). About 22% of adults with asthma had no ACEs, 19% had one ACE, 14% had two ACEs, and 45% had three or more ACEs.

There was a significant bivariate association between asthma severity and age, sex, race, mold in the home, smoking status, and income. The proportion of adults who reported persistent asthma was greater than the proportion who reported intermittent asthma among the following groups: women (51.7% vs. 48.3%), adults aged 45–54 years (52.6% vs. 47.4%), adults aged 55–64 years (52.8% vs. 47.2%), adults aged ≥ 65 years (53.3% vs. 46.7%), having mold in the home (53% vs. 47%), household with income < USD 25,000 (52.6% vs. 47.4%), and current smokers (50.4% vs. 49.6).

### 3.2. The Association Between ACEs and Asthma Severity

A one-unit increase in ACEs score was associated with a 5.4% increase in the odds of persistent asthma (adjusted odds ratio, aOR = 1.054 (95% confidence interval, CI = 1.01–1.10) (Table 2).

Experiencing ≥ 3 ACEs compared to no ACEs was associated with 31% increased odds of persistent asthma (aOR = 1.31, 95% CI = 1.01–1.70).

**Figure 1:**
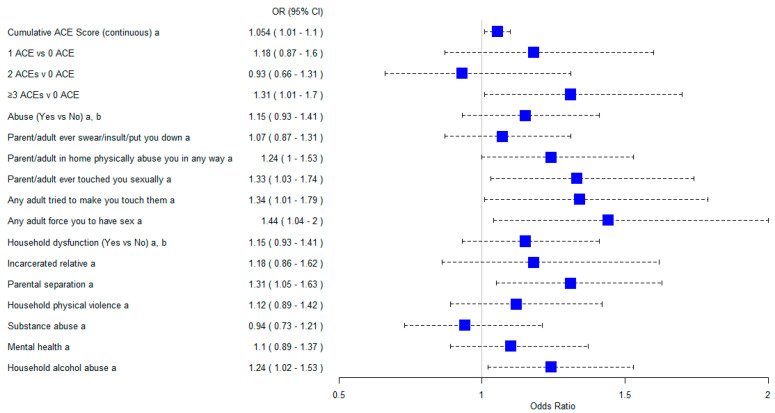
CI, confidence interval; OR, odds ratio; ACEs, adverse childhood experiences. ^a^ Adjusted for age, sex, race, income, mold in the home, smoking status, household smoke exposure, and baseline comorbidity. ^b^ Composite variable created from individual item ACE.

Individual ACE items significantly associated with persistent asthma include; parent/adult ever touched you sexually (aOR = 1.33, 95% CI = 1.03–1.74), adult tried to make you touch them (aOR = 1.34, 95% CI = 1.01–1.79), any adult forced you to have sex (aOR = 1.44, 95% CI = 1.04–1.20), parental separation/divorce (aOR = 1.31, 95% CI = 1.05–1.63), and household alcohol abuse (aOR = 1.24, 95% CI = 1.01–1.53) (Figure 1).

### 3.3. The Association Between ACEs and Asthma Severity Stratified by Gender

**Figure 2:**
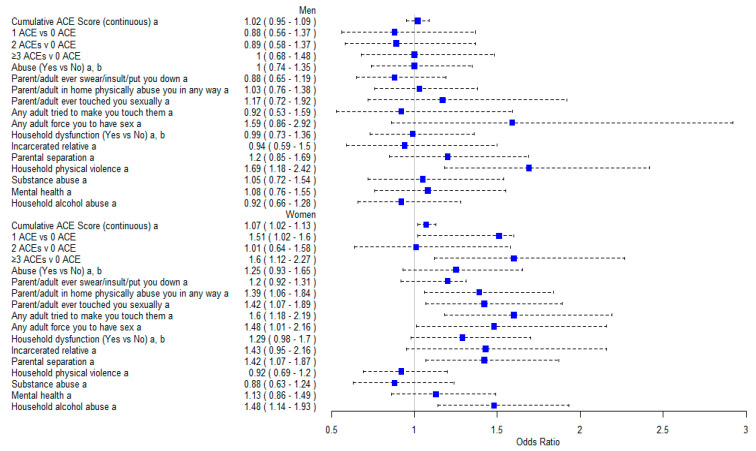
CI, confidence interval; OR, odds ratio; adverse childhood experiences. ^a^ Adjusted for age, race, income, mold in the home, smoking status, household smoke exposure, and baseline comorbidity. ^b^ Composite variable created from individual item ACE.

In women, a one-unit increase in ACE score was associated with a 7% increase in the odds of persistent asthma (aOR = 1.07, CI = 1.02–1.13) (Figure 2. Experiencing one ACE and ≥three ACEs (compared to no ACEs) was associated with 51% and 60% increased odds of having persistent asthma, respectively (aOR = 1.51, 95% CI = 1.02–2.23; aOR = 1.60, 95% CI = 1.12–2.27) in women. Individual ACE items significantly associated with persistent asthma in women include; physical abuse (aOR = 1.39, 95% CI = 1.06–1.84), parent/adult ever touched you sexually (aOR = 1.42, 95% CI = 1.071.89), adult tried to make them touch them (aOR = 1.60, 95% CI = 1.18–2.19), any adult force you to have sex (aOR = 1.48, 95% CI = 1.01–2.16), parental separation (aOR = 1.42, 95% CI = 1.07–1.87), and household alcohol abuse (aOR = 1.48, 95% CI = 1.48–1.93) (Table 2). No significant association was observed between ACEs and asthma severity in men; however, experiencing household physical violence (compared to no household physical violence experience) was associated with persistent asthma in men (aOR = 1.69, 95% CI = 1.18–2.42).

## 4. Discussion

Our findings showed that an increased ACE score is associated with an increased likelihood of persistent asthma in adults. Further, endorsing three or more ACEs was associated with higher odds of persistent asthma, implying the additive role ACEs may play in asthma severity. Indeed, as has already been shown, ACEs tend to cooccur rather than exist in isolation [31]. Among the ACEs that were found to convey the highest risk of adulthood asthma severity, sexual harassment, forced sex, parental separation, and alcohol abuse were the ones with the strongest association. This observation agrees with previous research supporting that childhood adversity could contribute to chronic health conditions [14]. A plausible explanation supporting this thesis is that the victims of childhood adversity tend to adopt risky health behaviors (e.g., smoking) that increase the risk—and possibly the severity—of developing chronic diseases in adulthood, including asthma [32]. Apart from this behavioral aspect, research has revealed that stress derived from experiencing ACEs might contribute to chronic health conditions [33]. The hyperactivity of the hypothalamus–pituitary–adrenal (HPA) axis and the resistance to cortisol-mediated negative feedback makes ACE-affected persons carriers of a heavy allostatic load that can be translated into adverse health outcomes later in life [34,35]. The above statement has been validated in animal models by Chida et al. (2007) [36]. Importantly, another study has shown that children who experience ACEs have lower expression of beta-2 glucocorticoid receptors and are more resistant to treatment with beta-2 agonists [37], possibly increasing the risk of persistent asthma in adulthood. There is also some evidence supporting that childhood adversity might be associated with pro-inflammatory phenotypes, as reflected by increased levels of inflammation mediators, such as C-reactive protein (CRP) [38].

ACEs have also been associated with biological senescence and shortening of telomere length [39], with detrimental effects on respiratory health [40]. This opens new questions as to whether adversity can cross generations and be transmitted to the offspring of the affected ones [41]. An interesting systematic review provides insight into how childhood adversity can influence one’s epigenetic signature in asthma [42]. This paper further supports the hypothesis of the intergenerational effect ACEs can have since epigenetic signatures can be passed to the offspring through the phenomenon of imprinting [43].

What is of particular interest when interpreting our results is that the association between ACEs and asthma severity differed between men and women. This finding aligns with what has already been observed regarding gender differences in ACEs [44]. On the one hand, women were found to have a 7% increase in the chances of having persistent asthma per ACE score unit, and ACEs were especially prominent in women who experienced either one (compared to no ACEs) or three or more ACEs (compared to no ACEs). Physical and sexual abuse, as well as parental divorce, were the factors that carried the highest risk of persistent asthma in women, highlighting the gender-specific impact of ACEs in adulthood asthma. On the other hand, the association either of ACE score or categorical ACEs on asthma severity in men was not statistically significant. When assessing the specific ACEs that influence asthma severity in men, only household physical violence was found to be statistically significant. We attribute this gender discrepancy to three main reasons. First, the sample size of women in our data was almost double than that of men. Hence, the analysis of data regarding men might have been more prone to random error, and what was observed in women may be closer to what is true in the population when evaluating larger sample sizes. Second, there have been studies that support that women are more likely to be the victims of childhood adversity [45], and to thereby withstand the negative consequences of ACEs in chronic health outcomes. Finally, there is evidence that supports that women might be more likely to report ACEs than men [46].

The fact that the persistent phenotype makes up 45.3% of US adults with asthma makes adulthood asthma a significant health issue. Since identifying persistent asthma is crucial for effective therapeutic interventions, an active search for ACEs should be an integral part of the history-taking process in the clinical setting. However, caution is required when implementing this approach in clinical practice, as the physician should take the actionability of such findings into account [47].

Our findings also raise the question as to whether addressing the burden ACEs raise helps manage adult patients with persistent asthma by mitigating the detrimental effects of toxic stress and inflammation. There is evidence revealing that targeted social support may be of benefit when attempting to control the health risks childhood adversity poses [48].

One of the primary strengths of this study is the use of a large sample of adults currently diagnosed with asthma. We evaluated the status of asthma severity within those states that contributed to the Asthma Call-Back Survey (ACBS). It is worth noting that ACBS is the sole survey that provides the majority of indicators necessary for classifying asthma severity and control in accordance with the guidelines established by the National Asthma Education and Prevention Program (NAEPP, EPR-3). This makes the ACBS a critical survey for evaluating asthma severity and control at the population level.

The results presented herein should be interpreted by considering some limitations of our study. A notable limitation of this study lies in the restrictions imposed by the available indicators within ACBS, which were used for classifying asthma severity.

The ACBS questionnaire content precluded the inclusion of all elements required by the National Asthma Education and Prevention Program (NAEPP) guidelines (activity limitation, pulmonary function measures, asthma exacerbations necessitating oral corticosteroids). This constraint could potentially lead to an underestimation of the prevalence of persistent asthma. Nevertheless, the classification of asthma severity in this analysis aligns with definitions used in other population-based estimates [49,50].

Regarding sample characteristics, an overrepresentation of female sex as well as white, non-Hispanic ethnic group was noted. In addition, a significant bivariate association between asthma severity and age, sex, race, mold in the home, smoking status, and income is observed, and a correlation between ACEs and these variables is possible and could be further explored in the future. Moreover, due to the observational, cross-sectional nature of this study, we cannot avoid recall bias nor establish a causal relationship between ACEs and asthma severity. Further, despite controlling key sociodemographic and environmental variables, residual confounding may still influence the observed association between ACEs and asthma severity. Longitudinal studies that follow victims of childhood adversity well into adulthood are expected to shed more light on this. Lastly, our findings cannot be generalized to adults with current asthma in states that did not participate in the ACBS or institutionalized patients.

## 5. Conclusions

In conclusion, our findings provide some evidence to suggest a significant association between ACEs and the severity of asthma in later life. The implications of these findings underscore the importance of preventive strategies and early interventions to reduce ACEs, with the aim of potentially mitigating the severity of asthma in US adults with asthma. Future research should aim to clarify the mechanisms underlying this association. These findings carry the potential to shift paradigms in our understanding of asthma and its management, providing new opportunities for holistic, patient-centered care that incorporates both physical and psychological components.

## Figures and Tables

**Table 1:** Descriptive statistics of US adults with asthma, 2019 and 2020 Asthma Call-Back Survey.

Characteristics			Asthma Severity			
	Survey Respondents		Intermittent Asthma		Persistent Asthma	
	No, ^a^	% ^b^ (95% CI ^b^)	No, ^a^	% ^b^ (95% CI ^b^)	No, ^a^	% ^b^ (95% CI ^b^)
**Total**	19,033		9445	54.7 (53.2–56.3)	9588	45.3 (43.7–46.8)
**ACEs score, mean (SD)** ^d^	7238	2.6 (2.5)	3517	2.43 (2.45)	3721	2.70 (2.65)
**ACEs** ^c^						
0	1830	21.6 (19.7–23.5)	902	57.1 (52.6–61.6)	928	42.9 (38.4–47.4)
1	1438	19.3 (17.6–21.1)	731	53.2 (48.3–58.2)	707	46.8 (41.8–51.7)
2	1016	14.3 (12.7–15.9)	539	58.8 (52.9–64.8)	477	41.2 (35.2–47.1)
≥3	2954	44.8 (42.5–47.1)	1345	50.7 (47.3–54.0)	1609	49.3 (46.0–52.7)
**Age** ^c^						
18–34 years	3965	37.8 (36.2–39.4)	2416	64.3 (61.7–67.0)	1549	35.7 (33.0–38.3)
35–44 years	2516	16.5 (15.3–17.7)	1366	54.0 (50.1–57.9)	1150	46.0 (42.1–49.9)
45–54 years	2832	15.1 (14.1–16.1)	1366	47.4 (43.8–51.0)	1496	52.6 (49.0–56.2)
55–64 years	3898	14.7 (13.8–15.7)	1686	47.2 (43.6–50.8)	2212	52.8 (49.2–56.4)
≥65 years	5822	15.9 (15.0–16.8)	2641	46.7 (43.7–49.7)	3181	53.3 (50.3–56.3)
**Gender** ^c^						
Women	12,070	59.3 (57.8–60.8)	5372	48.3 (46.2–50.3)	6698	51.7 (49.7–53.8)
Men	6963	40.7 (39.2–42.2)	4073	64.2 (62.0–66.4)	2890	35.8 (33.6–38.0)
**Race/ethnicity** ^c^						
White, Non-Hispanic	14,813	66.4 (64.9–68.0)	7252	52.7 (51.1–54.4)	7561	47.3 (45.6–48.9)
Black, Non-Hispanic	926	9.9 (8.8–11.0)	469	62.2 (56.7–67.6)	457	37.8 (32.4–43.3)
Hispanic	1668	16.6 (15.3–17.9)	886	55.7 (51.1–60.3)	782	44.3 (39.7–48.9)
Multiple/other race, Non-Hispanic	1354	7.0 (6.1–8.0)	703	60.7 (54.2–67.2)	651	39.3 (32.8–45.8)
**Mold in home** ^c^						
No	17,170	90.2 (89.2–91.1)	8680	55.7 (54.1–57.2)	8490	44.3 (42.8–45.9)
Yes	1787	9.8 (8.9–10.8)	738	47.0 (41.8–52.2)	1049	53.0 (47.8–58.2)
**Smoking status** ^c^						
Never smokers	11,841	65.0 (63.5–66.4)	5937	56.5 (54.6–58.4)	5904	43.5 (41.6–45.4)
Former smokers	4980	22.5 (22.1–23.7)	2484	52.3 (49.3–55.3)	2496	47.7 (44.7–50.7)
Current smokers	2118	12.5 (11.6–13.5)	970	49.6 (45.6–53.7)	1148	50.4 (46.3–54.4)
**Household smoke exposure**						
No	17,373	89.6 (88.7–90.5)	8666	54.9 (53.2–56.5)	8707	45.1 (43.5–46.8)
Yes	1646	10.4 (9.5–11.3)	774	54.0 (49.4–58.6)	872	46.0 (41.4–50.6)
**Household income** ^c^						
≥USD 25,000	12,422	74.9 (73.4–76.3)	6474	56.8 (54.9–58.6)	5948	43.2 (41.4–45.1)
<USD 25,000	4066	25.1 (23.7–26.6)	1725	47.4 (44.0–50.8)	2341	52.6 (49.2–56.0)
**Baseline comorbidity (mean ± SD)** ^d^	19,033	1.11 (1.17)	9445	0.99 (1.13)	9588	1.23 (1.19)
**Individual ACE components**						
**Abuse**						
No	2986	39.2 (37.0–41.4)	1518	56.4 (53.0–59.8)	1468	43.6 (40.2–47.0)
Yes	4246	60.8 (58.6–63.0)	1996	52.0 (49.2–54.9)	2250	48.0 (45.1–50.8)
**Parent/adult ever swear/insult/put you down**						
No	3853	50.9 (48.7–53.2)	1932	54.8 (51.8–57.9)	1921	45.2 (42.1–48.2)
Yes	3299	49.1 (46.8–51.3)	1550	52.4 (49.2–55.6)	1749	47.6 (44.4–50.8)
**Parent/adult in home physically abuse you in any way**						
No	5573	75.6 (73.6–77.7)	2771	55.1 (52.6–57.5)	2802	44.9 (42.5–47.4)
Yes	1555	24.4 (22.3–26.4)	699	49.8 (45.0–54.6)	856	50.2 (45.4–55.0)
**Parent/adult ever touched you sexually** ^c^						
No	5688	80.2 (78.4–82.0)	2856	55.9 (53.5–58.3)	2832	44.1 (41.7–46.5)
Yes	1446	19.8 (18.0–21.6)	614	45.6 (40.4–50.8)	832	54.4 (49.2–59.6)
**Any adult tried to make them touch them** ^c^						
No	6105	86.0 (84.4–87.6)	3037	54.8 (52.5–57.2)	3068	45.2 (42.8–47.5)
Yes	1020	14.0 (12.4–15.6)	434	47.7 (41.6–53.7)	586	52.3 (46.3–58.4)
**Any adult force you to have sex** ^c^						
No	6504	90.4 (89.2–91.7)	3233	54.6 (52.3–56.9)	3271	45.4 (43.1–47.7)
Yes	638	9.6 (8.3–10.8)	240	45.1 (38.1–52.1)	398	54.9 (47.9–61.9)
**Household dysfunction**						
No	2912	34.5 (32.3–36.6)	1423	55.7 (52.2–59.2)	1489	44.3 (40.8–47.8)
Yes	4326	65.5 (63.4–67.7)	2094	52.7 (49.9–55.5)	2232	47.3 (44.5–50.1)
**Incarcerated relative**						
No	6472	85.8 (84.1–87.5)	3139	53.9 (51.5–56.2)	3333	46.1 (43.8–48.5)
Yes	739	14.2 (12.5–15.9)	363	52.3 (45.9–58.6)	376	47.7 (41.4–54.1)
**Parental separation**						
No	4916	60.3 (58.0–62.6)	2384	55.0 (52.3–57.6)	2532	45.0 (42.4–47.7)
Yes	2174	39.7 (37.4–42.0)	1069	52.3 (48.5–56.2)	1105	47.7 (43.8–51.5)
**Household physical violence** ^c^						
No	4839	65.9 (63.7–68.2)	2469	55.7 (53.0–58.3)	2370	44.3 (41.7–46.0)
Yes	2328	34.1 (31.8–36.3)	1018	50.4 (46.5–54.3)	1310	49.6 (45.7–53.5)
**Substance abuse**						
No	6099	81.6 (79.8–83.4)	2949	53.2 (50.8–55.5)	3150	46.8 (44.5–49.2)
Yes	1086	19.4 (16.6–20.2)	546	56.1 (50.7–61.4)	540	43.9 (38.6–49.3)
**Mental health**						
No	5026	67.1 (65.0–69.2)	2467	54.9 (52.3–57.6)	2559	45.1 (42.4–47.7)
yes	2149	32.9 (30.7–35.0)	1018	51.2 (47.3–55.1)	1131	48.8 (44.9–52.7)
**Household alcohol abuse** ^c^						
No	5047	67.8 (65.7–70.0)	2504	55.4 (52.8–58.0)	2543	44.6 (42.0–47.2)
Yes	2163	32.2 (30.0–34.3)	1001	50.2 (46.2–54.2)	1162	49.8 (45.8–53.8)

CI, confidence interval; ACEs, adverse childhood experience. ^a^: Unweighted pooled sample size. Due to item non-response, individual characteristic categories may not sum to total. ^b^: Weighted prevalence in %. ^c^: *p*-values < 0.05 for the chi-square test of association between outcome and variables. ^d^: *p*-values < 0.05 for t-test of mean difference.

**Table 2:** Multivariable logistic regression of the association between adverse childhood experiences (ACEs) before 18 years and asthma severity.

Variable	Unadjusted OR (95% CI)	*p*-Value	Adjusted OR (95% CI)	*p*-Value
**Cumulative ACE Score (continuous)** ^a^	1.12(1.02–1.22)	0.008	1.054 (1.01–1.10)	0.01
**ACE categories** ^a^				
0	Ref		Ref	
1 ACE	1.19 (0.91–1.57)	0.32	1.18 (0.87–1.60)	0.29
2 ACEs	0.93 (0.68–1.27)	0.19	0.93 (0.66–1.31)	0.66
≥ 3 ACEs	1.29 (1.08–1.63)	0.02	1.31 (1.01–1.70)	0.04
**Abuse (Yes vs. No)** ^a,b^	1.18 (0.98–1.42)	0.07	1.15 (0.93–1.41)	0.20
Parent/adult ever swear/insult/put you down ^a^	1.08 (0.90–1.30)	0.41	1.07 (0.87–1.31)	0.54
Parent/adult in home physically abuse you in any way ^a^	1.22 (1.01–1.49)	0.04	1.24 (1.00–1.53)	0.05
Parent/adult ever touched you sexually ^a^	1.50 (1.18–1.90)	0.001	1.33 (1.03–1.74)	0.03
Any adult tried to make them touch them ^a^	1.32 (1.01–1.73)	0.04	1.34 (1.01–1.79)	0.04
Any adult force you to have sex ^a^	1.42 (1.05–1.92)	0.02	1.44 (1.04–2.00)	0.03
**Household dysfunction (Yes vs. No)** ^a,b^	1.13 (0.94–1.36)	0.19	1.15 (0.93–1.41)	0.20
Incarcerated relative ^a^	1.04 (0.79–1.38)	0.76	1.18 (0.86–1.62)	0.29
Parental separation ^a^	1.11 (0.92–1.35)	0.28	1.31 (1.05–1.63)	0.02
Household physical violence ^a^	1.24 (1.00–1.54)	0.05	1.12 (0.89–1.42)	0.33
Substance abuse ^a^	0.88 (0.69–1.12)	0.30	0.94 (0.73–1.21)	0.63
Mental health ^a^	1.15 (0.95–1.40)	0.15	1.10 (0.89–1.37)	0.38
Household alcohol abuse ^a^	1.22 (1.09–1.49)	0.04	1.24 (1.02–1.53)	0.04

CI, confidence interval; OR, odds ratio; ACEs, adverse childhood experiences. ^a^ Adjusted for age, sex, race, income, mold in the home, smoking status, household smoke exposure and baseline comorbidity. ^b^ Composite variable created from component ACE variables in bullet points.

## Data Availability

The data used to generate the findings of this study are publicly available from the Asthma Call-Back Survey Website available at: https://www.cdc.gov/brfss/acbs/index.htm (accessed on 9 September 2024). The Asthma Call-Back Survey (ACBS) is a product of the CDC’s National Asthma Control Program (NACP).

## Supplementary Materials

The following supporting information can be downloaded at: https://www.mdpi.com/article/10.3390/medsci12040063/s1, Table S1: Classification of asthma control in adults modified from the National Asthma Education and Prevention Program Expert Panel Report 3 Guidelines. 23. Table S2: Classification of asthma severity for research and population-based estimates from the National Asthma Education and Prevention Expert Panel Report 3 guidelines 23. Table S3: Behavioral Risk Factor Surveillance System (BRFSS) Adverse Childhood Experiences Survey Item.
