# Supplementary material for: The Impact of Adverse Childhood Experiences on Asthma Severity in US Adults
Source: Med Sci (Basel). 2024 Nov 11;12(4):63. doi: 10.3390/medsci12040063 (PMC11587021; doi:10.3390/medsci12040063)
Supplement: Supplementary file 1 [file medsci-12-00063-s001.zip › medsci-3230166-supplementary.pdf]

medical sciences

MDPI

Supplementary Material

Table S1. Classification of asthma control in adults modified from the National Asthma Education and Prevention Program Expert Panel Report 3 Guidelines. [23].

This table modifies the classification from the National Asthma Education and Prevention Program Expert Panel Report 3 Guidelines. It shows the criteria for controlled, uncontrolled (not well controlled, very poorly controlled) asthma based on symptoms, night-time awakenings, and short-acting β2-agonist use.

Measures of current impairment	Controlled	Uncontrolled	
	Well controlled	Not well controlled	Very poorly controlled
Symptoms	≤2 d/ week	>2 d/ week	Throughout the day
Night-time awakenings			
Ages 12 years or older	≤2 times/month	> 1–3 times/ week	≥4 times/week
Short-acting β2-agonists used for symptom control	≤2 d/week	> 2d/ week	Several times/day

[23] Zahran HS, Bailey CM, Qin X, Moorman JE. Assessing asthma severity among children and adults with current asthma. J Asthma. 2014;51(6):610-617. doi:10.3109/02770903.2014.892966.

Table S2. Classification of asthma severity for research and population-based estimates from the National Asthma Education and Prevention Expert Panel Report 3 guidelines [23].

Adapted from the National Asthma Education and Prevention Expert Panel Report 3 guidelines, this table classifies asthma severity into intermittent and persistent categories, further divided based on long-term control medication use and asthma control status.

Asthma severity status	Long-term control medication use	Asthma control status
Intermittent asthma	No	Well controlled
Persistent asthma	Yes	Well controlled
		Not well controlled
		Very poorly controlled asthma
	No	Not well controlled or very poorly controlled asthma

[23] Zahran HS, Bailey CM, Qin X, Moorman JE. Assessing asthma severity among children and adults with current asthma. J Asthma. 2014;51(6):610-617. doi:10.3109/02770903.2014.892966.

Table S3. Behavioral Risk Factor Surveillance System (BRFSS) Adverse Childhood Experiences Survey Item.

This table lists the specific items from the Behavioral Risk Factor Surveillance System (BRFSS) ACEs survey used in our analysis, including the type of ACE each item represents. It provides a comprehensive overview of the ACE variables measured, such as household mental illness, substance abuse, and physical or emotional abuse, among others.

Item	Content	ACE type
1	Did you live with anyone who was depressed, mentally ill, or suicidal?[a]	Household Mental Illness
2	Did you live with anyone who was a problem drinker or alcoholic?[a]	Household Alcohol Abuse
3	Did you live with anyone who used illegal street drugs or who abused prescription medications?[a]	Household Substance Abuse
4	Did you live with anyone who served time or was	Incarcerated Family Member

	sentenced to serve time in a prison, jail, or other correctional facility?[a]	
5	Were your parents separated or divorced?[a]	Parental Separation/Divorce
6	How often did your parents or adults in your home ever slap, hit, kick, punch or beat each other up?b	Household Physical Violence
7	Before age 18, how often did a parent or adult in your home ever hit, beat, kick, or physically hurt you in any way?[b]	Physical Abuse
8	How often did a parent or adult in your home ever swear at you, insult you, or put you down?[b]	Emotional Abuse
9	How often did anyone at least 5 years older than you or an adult, ever touch you sexually?[b]	Sexual Abuse
10	How often did anyone at least 5 years older than you or an adult, try to make you touch them sexually?[b]	Sexual Abuse
11	How often did anyone at least 5 years older than you or an adult, force you to have sex?[b]	Sexual Abuse

a Dichotomous scale—yes/no.
b Likert scale—none, once, more than once. Recoded as dichotomous (none, once/more than once) for this analysis.
